# Predicting mortality in brain stroke patients using neural networks: outcomes analysis in a longitudinal study

**DOI:** 10.1038/s41598-023-45877-8

**Published:** 2023-10-28

**Authors:** Nasrin Someeh, Mani Mirfeizi, Mohammad Asghari-Jafarabadi, Shayesteh Alinia, Farshid Farzipoor, Seyed Morteza Shamshirgaran

**Affiliations:** 1 Student Research Committee, Tabriz University of Medical Sciences, Tabriz, Iran; 2 Werribie Mercy West Hospital, Werribee, VIC 3030 Australia; 3 Road Traffic Injury Research Center, Tabriz University of Medical Sciences, Tabriz, Iran; 4 Cabrini Research, Cabrini Health, Malvern, VIC 3144 Australia; 5 School of Public Health and Preventative Medicine, Faculty of Medicine, Nursing and Health Sciences, Monash University, Melbourne, VIC, 3004 Australia; 6 Department of Psychiatry, School of Clinical Sciences, Faculty of Medicine, Nursing and Health Sciences, Monash University, Clayton, VIC 3168 Australia; 7 Department of Biostatistics and Epidemiology, School of Medicine, Zanjan University of Medical Sciences, Zanjan, Iran; 8 Department of Statistics and Epidemiology, Faculty of Health, Tabriz University of Medical Sciences, Tabriz, Iran; 9 Department of Statistics and Epidemiology, Faculty of Health Sciences, Neyshabur University of Medical Sciences, Neyshabur, Iran

**Keywords:** Epidemiology, Machine learning, Statistical methods, Statistics, Neuroscience, Health care, Risk factors, Mathematics and computing

## Abstract

In this study, Neural Networks (NN) modelling has emerged as a promising tool for predicting outcomes in patients with Brain Stroke (BS) by identifying key risk factors. In this longitudinal study**,** we enrolled 332 patients form Imam hospital in Ardabil, Iran, with mean age: 77.4 (SD 10.4) years, and 50.6% were male. Diagnosis of BS was confirmed using both computerized tomography scan and magnetic resonance imaging, and risk factor and outcome data were collected from the hospital’s BS registry, and by telephone follow-up over a period of 10 years, respectively. Using a multilayer perceptron NN approach, we analysed the impact of various risk factors on time to mortality and mortality from BS. A total of 100 NN classification algorithm were trained utilizing STATISTICA 13 software, and the optimal model was selected for further analysis based on their diagnostic performance. We also calculated Kaplan–Meier survival probabilities and conducted Log-rank tests. The five selected NN models exhibited impressive accuracy ranges of 81–85%. However, the optimal model stood out for its superior diagnostic indices. Mortality rate in the training and the validation data set was 7.9 (95% CI 5.7–11.0) per 1000 and 8.2 (7.1–9.6) per 1000, respectively (P = 0.925). The optimal model highlighted significant risk factors for BS mortality, including smoking, lower education, advanced age, lack of physical activity, a history of diabetes, all carrying substantial importance weights. Our study provides compelling evidence that the NN approach is highly effective in predicting mortality in patients with BS based on key risk factors, and has the potential to significantly enhance the accuracy of prediction. Moreover, our findings could inform more effective prevention strategies for BS, ultimately leading to better patient outcomes.

## Introduction

Brain stroke is a significant global health concern, with a high mortality rate and permanent disability incidence^1,2^. According to the World Health Organization, approximately 15 million people worldwide suffer from brain stroke each year, resulting in 5 million deaths and 5 million permanent disabilities^3^. Over the years, the incidence, prevalence, and related mortality and disability-adjusted life years have shown an alarming increase, emphasizing the urgent requirement for targeted prevention and healthcare strategies^1,2,4^.

Understanding risk factors is crucial for planning evidence-based BS care and allocating resources effectively. In 2019, the primary BS risk factors included high systolic blood pressure (contributing to 55.5% of total stroke Disability-Adjusted Life Years (DALYs)), high body mass index (24.3%), high fasting plasma glucose (20.2%), and smoking (17.6%)^1^. Hypertension is the most significant risk factor for BS^2,5–7^, while a history of hyperlipoproteinemia and diabetes increases the risk of cardiovascular and cerebrovascular diseases, including ischemic BS^8^. Smoking and passive smoking have been identified as leading risk factors for BS in several studies^9,10^, with passive smoking increasing the risk of BS by 30%^11^. In Iran, 44.7% of national stroke ASDRs were linked to hypertension, while 28.8% were attributed to high fasting plasma glucose^4^. Addressing these risks through targeted measures and screenings can be crucial for prevention.

Predicting BS mortality, as a most destructive and prevalent nervous system diseases worldwide, is crucial for healthcare planning. Besides conventional statistics like logistic regression and survival analysis, machine learning and data-driven approaches including decision trees, random forest, support vector machines, k-nearest neighbours, naive bayes, gradient boosting algorithms, deep learning, and neural networks have been used to predict the mortality. These algorithms can make better predictions by using multiple variables at once and harnessing machine learning's strengths, including handling complex relationships, flexibility, automatic feature selection, managing imbalanced data, non-parametric approaches, and continuous learning^12^. There are some studies utilized deep learning algorithm^13–15^, particularly Neural Networks (NN) to predict the BS mortality^16,17^.

The evolving integration of NN into the medical field represents a pivotal avenue for exploring intricate nonlinear data relationships, enhancing data interpretation capabilities, and devising more effective diagnostic and predictive tools. Neural networks, inspired by the structural and functional attributes of the human neural architecture, have demonstrated the capacity to discern complex associations between input and output variables through iterative learning and validation procedures. Consequently, NN methodologies have gained increasing prominence in diverse facets of medical diagnosis and prediction, signifying a transformative influence within the healthcare domain^18^. As NN endeavours to emulate human cognitive functions, its ascension is propelled by the burgeoning availability of healthcare data and the rapid evolution of analytical techniques. NN exhibits adaptability in accommodating various forms of healthcare data, both structured and unstructured, with substantial utilization observed in prominent medical domains such as cancer, neurology, and cardiology. This review particularly underscores NN's pivotal role in the realm of stroke, spanning critical domains of early detection, diagnostic assessment, treatment optimization, and the precise evaluation of outcome prediction and prognosis^19^.

Existing research is primarily derived from limited preliminary studies characterized by a significant risk of bias and considerable heterogeneity as indicated by a recent systematic review of 28 studies employing ML models to predict BS mortality. Emphasizing the crucial aspect, ML algorithms can carry bias tied to factors like patient sampling, missing data, sample size, and misclassification error. While there is evidence supporting the potential superiority of the NN strategy in predicting individual patients with BS, it is important to note that such superiority may not always be consistent^20^. Besides, in deciding upon a tailored methodology, a careful assessment of the trade-offs between accuracy and the ease of interpretation becomes essential highlighting the need for comprehensible insights and thoughtful agreement^21^. Additionally, the GBD 2019 assessment, encompassing 204 countries and territories from 1990 to 2019, highlights the need for tailored strategies to address stroke burden variations influenced by specifically, geographical disparities and risk factors^1^ even in a subnational level within the country^4^. Hence, in light of the trade-offs inherent in employing NN algorithms and acknowledging geographical disparities, the primary objective of this study was to predict BS mortality by employing the most suitable NN methodology for modelling the latent risk factors. Additionally, the study aimed to conduct a comparative analysis of multiple classification-based NN models through the utilization of diagnostic indices.

## Materials and methods

### Study design and procedure

This longitudinal study collected data from the BS registry of the Imam Hospital, Ardabil, Iran, over a ten-year follow-up period from June 2008 to June 2018. The study included 332 patients who presented with first-time BS and were diagnosed using the International Coding System ICD-10 in accordance with both computerized tomography (CT) scan and magnetic resonance imaging (MRI).

### Main variables and measures

In this study, both demographic and clinical information were collected from hospital records for all patients. The demographic risk factors included age at diagnosis (categorized as 1: > = 58, 2: 59–68, 3: 69–75, < = 76), sex (categorized as 1: male, 2: female), employment status (categorized as 1: employed, 2: unemployed), place of residence (categorized as 1: urban, 2: rural), education level (categorized as 1: diploma-, 2: academic), smoking status (categorized as 1: yes, 2: no), former smoking (categorized as 1: yes, 2: no), waterpipe smoking (categorized as 1: yes, 2: no). Moreover, the clinical risk factors included the history of heart disease (categorized as 1: yes, 2: no), diabetes status (categorized as 1: yes, 2: no), oral contraceptive pill use (categorized as 1: yes, 2: no), physical activity (categorized as 1: yes, 2: no), history of stroke type (categorized as 1: ischemic, 2: haemorrhagic), history of high blood pressure (categorized as 1: yes, 2: no), history of hyperlipoproteinemia (categorized as 1: yes, 2: no), and history of myocardial infarction (categorized as 1: yes, 2: no). Notably, our data collection relied on comprehensive hospital registry information, ensuring the absence of exclusions due to insufficient data. Remarkably, we encountered no instances of missing risk factor data throughout our study.

In addition, we obtained survival outcome information through telephone follow-up over a follow-up period of 10 years. We tracked patients from their acute BS hospitalization until either they passed away or the follow-up period ended, whichever happened first. Also, we did not come across any cases of missing outcome data in our study.

### Inclusion and exclusion criteria

We included all patients with reported BS-related deaths in our study. Excluding cases where death was attributed to reasons other than BS, which was confirmed through family communication during follow-up, left us with a total of 78 excluded patients.

### Ethics approval and consent to participate

The study protocol was approved by the institutional review board of Tabriz University of Medical Sciences under ethics code IR.TBZMED.REC.1400.1142. This study was conducted in accordance with the principles of the Helsinki Declaration. Participants were given the freedom to choose whether or not to participate, and their privacy was protected. All participants provided written consent and filled out an informed consent form.

### Neural networks approach

**Figure 1:**
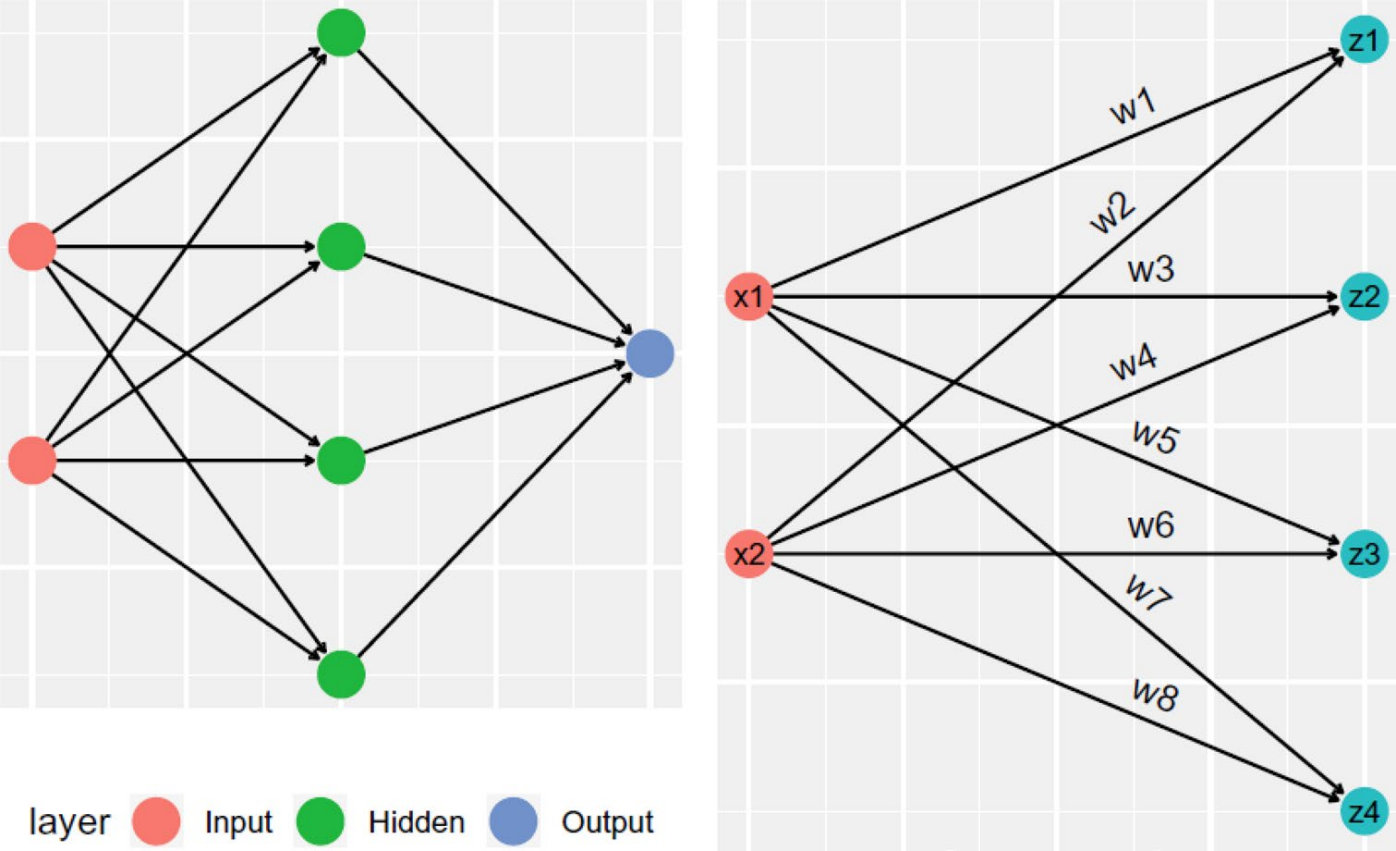
The basic neural network (NN) model. Left: A simple NN model with an input layer, a hidden layer, and an output layer. Right: The hidden layer computes the summation of the input and the weights and the bias, and calculates activation function.

The methodology for predicting BS mortality involved the utilization of NN. This process comprised two distinct datasets: a training dataset, constituting 70% of the total data, and a testing dataset, accounting for the remaining 30%. The allocation of patients into the training and validation sets was performed using a randomized approach. Specifically, we employed the randomization scheme integrated within the STATISTICA12 (Stat Soft, Statistica, Toluca, USA) software for this purpose. The primary purpose of the training dataset was to establish a predictive model through the application of a classification algorithm, while the testing dataset was employed to evaluate the model's classification efficacy. The NN architecture consisted of three layers: an input layer, a hidden layer, and an output layer (as depicted in Fig. 1). Within the input layer, pertinent variables were incorporated, with the hidden layer's nodes determining the output. The activation function, computed in the hidden layer, was subsequently transmitted to the output layer to generate the predicted outcomes. Evaluation of model performance and quality encompassed a range of diagnostic indices including sensitivity, specificity, positive predictive value, negative predictive value, accuracy, and the area under the receiver operating characteristic (ROC) curve. All metrics were accompanied by 95% confidence intervals (CIs). The adopted neural network framework was based on the multilayer perceptron (MLP) model. During the network's training phase, specific error functions like square and cross-entropy were employed to enhance its learning process. Various activation functions, such as the identity function, hyperbolic tangent, logistic sigmoid, exponential, sine, and softmax, were also utilized to tailor the model's performance to different requirements. A number of 18 key input features (variables) encompassed demographic and clinical information (18 variables as mentioned before in the “Main variables and measures” section). Furthermore, the incorporation of the Automated Network Search (ANS) method enriched the predictive capabilities of our models. This involved assessing a hundred trained networks, from which five optimal models were recommended based on their performance in both training and testing phases. Ultimately, we selected a single optimal NN model, which we presented through a radar plot generated using Microsoft Excel (Microsoft Corporation Rosa, California, USA). The significance of predictor variables was determined based on their importance weights. Variables with normalized weights exceeding 30% were categorized as high importance, those falling within the range of 10% to 30% were considered to have moderate importance, and variables with weights below 10% were classified as having low importance in predicting BS mortality. NN models were trained using STATISTICA software [ver.12] (Stat Soft, Statistica, Toluca, USA).

### Statistical analyses

Statistical analysis was performed using STATISTICA software [ver.12] (Stat Soft, Statistica, Toluca, USA). Numeric variables were expressed as mean (SD) or median (Percentile25–Percentile75), and categorical variables were expressed as frequency (percent). We utilized Fisher’s exact test to compare the percentages between survived and dead outcomes. Kaplan Meier method was used for classical survival analysis to calculate survival probabilities, and Log-rank tests were used to compare probabilities across groups. P-values < 0.05 were considered as significant.

## Results

**Figure 2:**
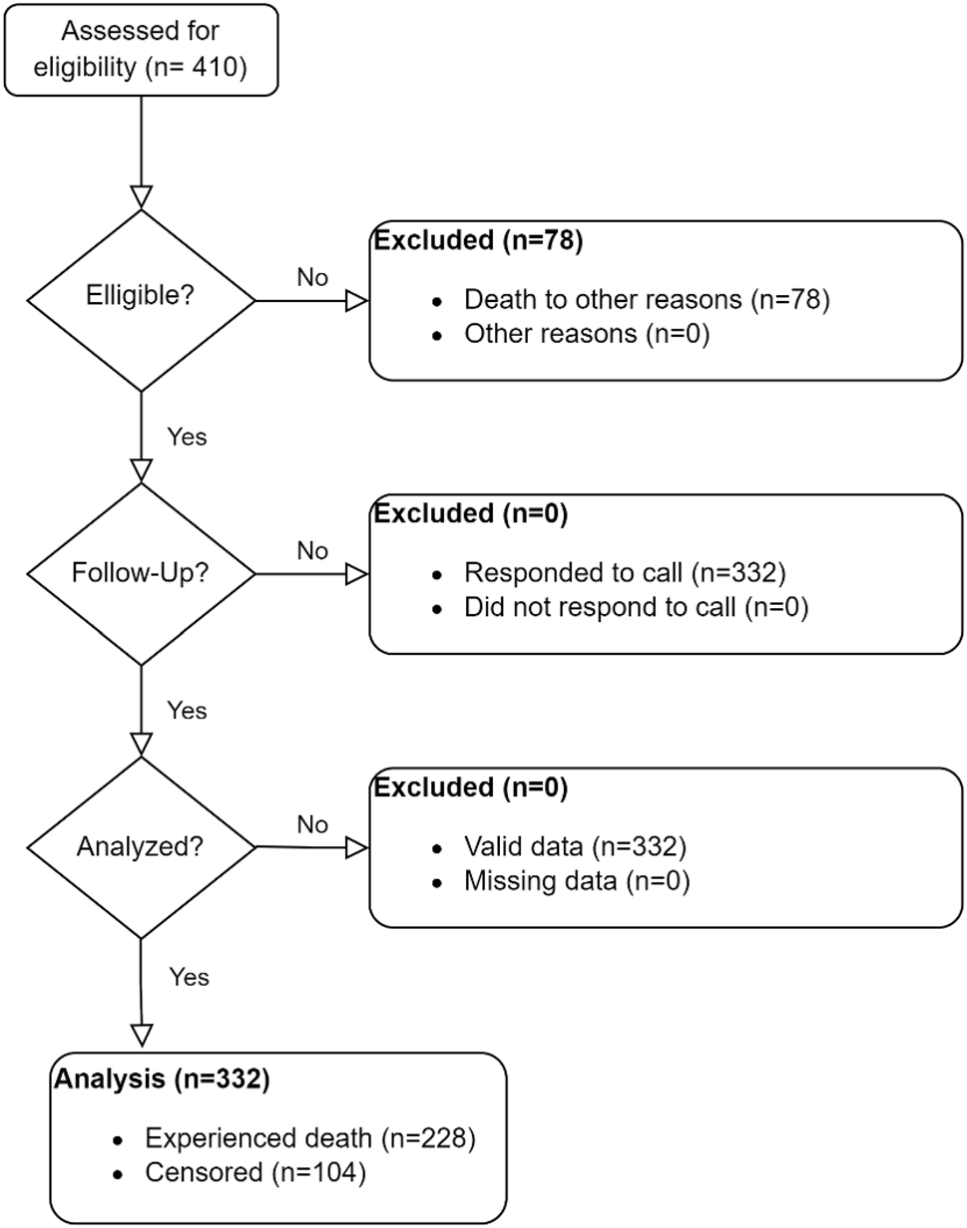
Study fellow diagram.

A total of 410 patients were enrolled in this study, out of which 332 were eligible for participation. The mean age of the participants was 67.2 (SD = 10.22) years, and 168 (50.6%) of participants were male. Over a follow-up period of 10 years, 228 (68.7%) patients experienced mortality (Fig. 2). The median follow-up time was 77.42 months (Percentile25 = 1.81, Percentile75 = 158.07).

The findings highlight notable associations, such as higher ages (P < 0.001), increased female representation (P = 0.013) and history of hyperlipoproteinemia (P = 0.022) being linked to higher death rates. Conversely, lower usage of oral contraceptive pills among women was associated with elevated death rates (P < 0.001) (Table 1).

**Table 1:** Demographic and clinical characteristics of the participants across survival and death outcomes.

Characteristic	Total outcome n (%)	Survival outcome n (%)	Death outcome n (%)	P-value
Age category (years)				**< 0.001**^**F**^
< = 58	88 (26.7)	57 (64.8)	31 (35.2)	
59–68	77 (23.3)	28 (36.4)	49 (63.6)	
69–75	102 (30.9)	14 (13.7)	88 (86.3)	
76+	63 (19.1)	5 (7.9)	58 (92.1)	
Sex				**0.013**^**F**^
Female	164 (49.4)	62 (37.8)	102 (62.2)	
Male	168 (50.6)	42 (25.0)	126 (75.0)	
Job				0.530^F^
Employed	107 (32.2)	36 (33.6)	71 (66.4)	
Unemployed	225 (67.8)	68 (30.2)	157 (69.8)	
Education				0.296^F^
Diploma or lower	332 (97.1)	99 (30.8)	223 (69.3)	
Academic	10 (2.9)	5 (50.0)	5 (50.0)	
Physical activity				0.235^F^
Yes	46 (13.9)	18 (39.1)	28 (60.9)	
No	284 (86.1)	86 (30.3)	198 (69.7)	
Smoking				0.454^F^
Yes	64 (19.3)	23 (35.9)	41 (64.1)	
No	267 (80.7)	81 (30.3)	186 (69.7)	
History of cerebrovascular				0.213^F^
Yes	80 (24.1)	30 (37.5)	50 (62.5)	
No	252 (75.9)	74 (29.4)	178 (70.6)	
History of myocardial infraction				0.261^F^
Yes	24 (7.2)	10 (41.7)	14 (58.3)	
No	308 (92.8)	94 (30.5)	214 (69.5)	
History of blood pressure				0.187^F^
Yes	196 (59.2)	56 (28.6)	140 (71.4)	
No	135 (40.8)	48 (35.6)	87 (64.4)	
History of heart disease				0.344^F^
Yes	85 (25.8)	23 (27.1)	62 (72.9)	
No	245 (74.2)	81 (33.1)	164 (66.9)	
History of diabetes				0.120^F^
Yes	59 (17.9)	13 (22.0)	46 (78.0)	
No	270 (82.1)	90 (33.3)	180 (66.7)	
History of hyperlipoproteinemia				**0.022**^**F**^
Yes	61 (18.5)	27 (44.3)	34 (55.7)	
No	269 (81.5)	77 (28.6)	192 (71.4)	
Cerebrovascular type				0.657^F^
Ischemic	257 (79.6)	84 (32.7)	173 (67.3)	
Hemorrhagic	66 (20.4)	19 (28.8)	47 (71.2)	
Oral contraceptive pill use (in women)				**< 0.001**^**F**^
Yes	60 (36.4)	35 (58.3)	25 (41.7)	
No	105 (63.6)	27 (25.7)	78 (74.3)	

F: Fisher’s exact test for comparing survival and death outcomes.

P-values for significant results are shown in bold.

**Figure 3:**
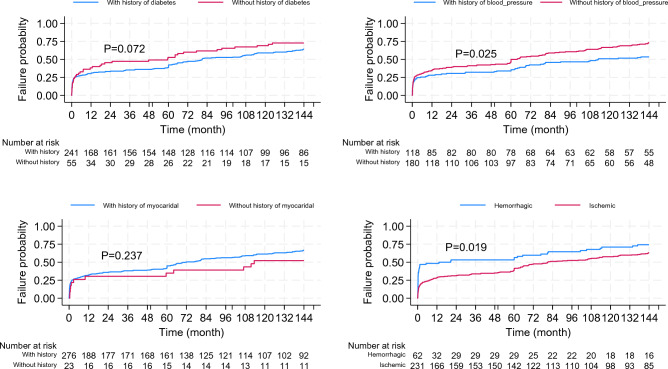
The probability of death in patients with Brain Stroke across some important risk factors. P-values were computed using log-rank test.

Results from Kaplan Meier failure rates and log-rank tests indicated that patients with a history of diabetes (P = 0.072), and myocardial disease (P = 0.237) had a higher risk of mortality but the differences were not significant. However, history of blood pressure (P = 0.025), and ischemic cerebrovascular disease (P = 0.019) were significantly associated with a higher risk of mortality (Fig. 3).

Additionally, a history of cerebrovascular disease (P = 0.295), and no physical activity (P = 0.061) had a higher risk of mortality but the differences were not significant. Nevertheless, being male (P = 0.017), older age (P < 0.001), history of hyperlipoproteinemia (P = 0.025), and oral contraceptive pill uses in females (P < 0.001) were significantly associated with a higher risk of mortality.

### Neural network modeling

The study explored different scenarios for the number of layers, with a minimum of 5 and a maximum of 17 hidden units being chosen as optimal. The output activation function selected was hyperbolic tangent. The study selected five final NN models. The diagnostic indices used to evaluate the models showed that sensitivity ranged from 93.8% (95% CI 87.5–97.5) to 96.4% (95% CI 91.9–99.0), specificity ranged from 94.8% (95% CI 87.2–98.6) to 100% (95% CI 93.2–100.0), positive predictive values ranged from 97.3% (95% CI 93.2–99.3) to 100% (95% CI 96.6–100.0), negative predictive values ranged from 85.9% (95% CI 76.6–92.5) to 92.9% (95% CI 82.7–98.0), area under the ROC curves ranged from 94.0 (95% CI 90.0–97.0) to 98.0 (95% CI 97.0–100.0), and model accuracies ranged from 81% (95% CI 78.4–83.6) to 85% (95% CI 82.7–87.3). All five models demonstrated a good fit, with the optimal model (MLP 40-9-2) showing the highest values for all diagnostic indices (Table 2).

**Table 2:** Results of comparing the retained neural network models by automated network search.

Model	SE% (95% CI)	SP% (95% CI)	Accuracy% (95% CI)	PPV% (95% CI)	NPV% (95% CI)	ROC% area
MLP 40-9-2	93.8 (87.5–97.5)	98.1 (89.7–100.0)	83.0 (80.5–85.5)	99.1 (94.9–100.0)	87.9 (76.7–95.0)	96.0 (93.0–99.0)
MLP 40-5-2	96.4 (91.1–99.0)	98.1 (98.7–100.0)	84.0 (81.6–86.4)	99.1 (95.0–100.0)	92.7 (82.4–98.0)	97.0 (95.0–100.0)
**MLP 40-9-2**	**96.4 (91.1–99.0)**	**100.0 (93.2–100.0)**	**85.0 (82.7–87.3)**	**100.0 (96.6–100.0)**	**92.9 (82.7–98.0)**	**98.0 (97.0–100.0)**
MLP 40-11-2	96.4 (91.1–99.0)	96.2 (86.8–99.5)	83.0 (80.5–85.5)	98.2 (93.6–99.8)	92.6 (82.1–97.9)	96.0 (93.0–100.0)
MLP 40-7-2	93.8 (87.5–97.5)	100.0 (93.2–100.0)	84.0 (81.6–86.4)	100.0 (96.5–100.0)	88.1 (77.1–95.1)	97.0 (95.0–100.0)
MLP 40-7-2	92.3 (86.9–95.9)	94.8 (87.2–98.6)	81.0 (78.4–83.6)	97.3 (93.2–99.3)	85.9 (76.6–92.5)	94.0 (90.0–97.0)

Optimal model is shown in bold.

*CI* confidence interval, *SE* sensitivity, *SP* specificity, *PPV* positive predictive value, *NPV* negative predictive value, *ROC* receiver operating characteristics.

**Figure 4:**
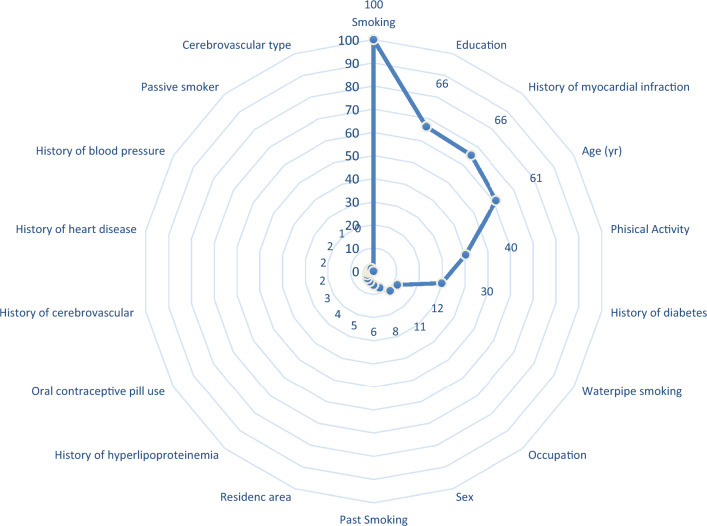
Radar plot for comparing the variable importance based on the optimal model. Normalized importance of the risk factors in brain stroke mortality prediction are displayed.

The significance of input variables' importance weights was visually represented using a radar plot. The optimal model elucidated substantial risk factors associated with BS mortality, notably including smoking, lower education, advanced age, physical inactivity, and a history of diabetes, all exhibiting high importance weights (> 30%). Furthermore, waterpipe smoking and occupation emerged as moderately influential risk factors, characterized by importance weights falling within the range of 10% to 30%. Conversely, other variables demonstrated comparatively lower importance weights (< 10%) in their role in predicting BS mortality (Fig. 4).

## Discussion

Stroke is a major public health issue that has significant consequences for individuals, families, and society as a whole^22^. This study showcases the application of the most critical statistical learning techniques of NN in predicting outcomes for patients with BS. ML methods^12,20^, particularly (deep learning) NN^12,13,15,17^, play a crucial role in disease prediction within the medical and health fields^20^. Researchers have utilized various ML techniques for diagnosing many diseases, including heart disease^23,24^, cancer^25^, specifically BS^26^, endorsing NN are a suitable candidate for mortality prediction^16,17,27^.

Similar to previous studies^24,28–30^, our approach involved optimizing the NN model through repeated trials with various NNs and assessing model quality using key indices^12,13,15,17,29^. The current study showcased remarkable findings in predicting BS mortality, encompassing high sensitivity (ranging from 93.8 to 96.4%), impressive specificity (ranging from 94.8 to 100%), robust positive predictive values (ranging from 97.3 to 100%), reliable negative predictive values (ranging from 85.9 to 92.9%), and substantial area under the ROC curves (ranging from 94.0 to 98.0). Notably, these results align with findings reported in other studies, highlighting the consistency and reliability of the predictive capabilities demonstrated in this study for BS mortality^17^. Chung et al. found a robust predictive model with an area under curve (AUC) of 0.976 for 3-month mortality prediction. The model exhibited excellent accuracy, sensitivity, and specificity values of 95.2%, 94.4%, and 95.5%, respectively, in predicting 3-month mortality after thrombolysis^17^. Cheon et al., conducted a study using deep learning to predict stroke patient mortality, yielding an AUC of 83.4, which was slightly lower compared to the results obtained in our study. Additionally, their study demonstrated that their deep learning NN outperformed various other ML methods^13^. A systematic review reported that out of 1015 retrieved studies, 28 were included, with 25 of them being retrospective. ML models showcased a favourable range of AUC for mortality prediction (0.67–0.98), with a predominant focus on short-term post-stroke mortality in most articles. Shanthi proposed using NN to model the outcomes in patients with BS, which proved to be an optimal prediction model with an overall predictive accuracy of 89%^27^. In another study^31^, the NN technique was presented for predicting heart disease by utilizing the genetic algorithm's global optimization advantage for initializing NN weights, achieving a classification accuracy of 89%. The number of explanatory features utilized in the models varied from 5 to 200, with significant overlap in the variables included^20^. Notably, in our study, by incorporating just 18 features, we achieved improved indices. These promising findings suggest the potential clinical utility of such models to inform decision-making when administering therapy.

In our study, the optimal model highlighted significant risk factors for BS mortality, including smoking, lower education, advanced age, lack of physical activity, a history of diabetes, waterpipe smoking, and being unemployed (occupation), all carrying substantial importance weights. However, it's important to note that despite the differences in study design and risk factors assessed, the results of various studies still demonstrate both similarities and dissimilarities. Furthermore, age, high BMI, and high NIHSS score were identified as important predictors for mortality in systematic review, further supporting our argument^20^. In another study^32^, utilizing Cox regression model, age, blood pressure, smoking, pre-existing cardiovascular disease, and diabetes were strongly associated with stroke mortality, which is consistent with some of the findings in the current study. However, some differences exist, such as blood pressure or pre-existing cardiovascular disease not being among the most influential risk factors in the current study. Another study^33^ that used a data mining approach to predict post-stroke mortality in different time scales found that age, socio-demographics, and past medical histories were the most significant risk factors for short/intermediate-term mortalities, which aligns with the current study's findings. Similarly, age, sex, hypertension, smoking, and alcohol consumption were the most prevalent risk factors in another study^34^, with only minor distinctions due to the unavailability of alcohol-related data in the current study. The results of Cox regression analysis in yet other studies^35,36^ identified age, sex, blood pressure, diabetes, hyperlipoproteinemia, and oral contraceptive pill as the most critical predictors for brain stroke, which is similar to the current study's findings in some risk factors. In contrast, a comprehensive global study conducted across 204 countries from 1990 to 2019 identified the leading risk factors for stroke as high systolic blood pressure (contributing to 55.5% of total stroke burden), high body-mass index (24.3% of total stroke burden), high fasting plasma glucose (20.2% of total stroke burden), ambient particulate matter pollution (20.1% of total stroke burden), and smoking (17.6% of total stroke burden). Though, our study's findings align with the global study in terms of the significance of smoking as a risk factor for BS mortality, the results differ from our study's focus on specific risk factors for BS mortality^1^. It's important to recognize that the risk factor profiles and their contributions to stroke and BS mortality can vary across different populations and regions.

### Strengths and limitations of the study

This study's strengths encompass the utilization of advanced statistical learning techniques, specifically NN, to predict outcomes in BS patients, thereby highlighting their clinical significance. Notably, it delves into specific risk factors, enriching the comprehension of underlying BS mortality mechanisms and providing insights for clinical decision-making. Nonetheless, certain limitations warrant consideration. The observational nature precludes causal inferences, urging exploration of causal modelling and hybrid approaches. Additionally, the dataset's origin from a single hospital registry and confined geographic area affects generalizability. Future studies should tap into larger and more diverse datasets, validate models across various populations, and explore interventions for predicted mortality risk reduction in BS patients. Another limitation pertains to the “black box” nature of NN, necessitating the development of precise statistical and ML methods to elucidate its real-world application^21^.

## Conclusion

In conclusion, this study advances our understanding of mortality prediction in patients with BS by leveraging the power of NN. The investigation of various risk factors and their contributions to BS mortality highlights the complexity of this multifaceted issue. Our findings contribute to the existing literature by providing a comprehensive analysis of significant risk factors and their importance weights in predicting BS mortality. The study's robust methodology and the demonstration of improved predictive indices using a relatively concise set of 18 features offer a valuable addition to the field of stroke research. Future research could delve deeper into the interplay of these risk factors, considering their interactions and potential regional variations. Moreover, exploring the integration of additional clinical, genetic, and environmental data could enhance the accuracy and applicability of mortality prediction models in clinical practice. This study paves the way for further investigations aimed at refining and tailoring mortality prediction strategies to individual patient profiles, thereby contributing to more targeted and effective interventions in stroke care.

## Data Availability

The datasets used and/or analysed during the current study are available from the corresponding author upon reasonable request.

## Abbreviations

NN: Neural networks
BS: Brain stroke
ML: Machine learning
MLP: Multilayer perceptron
ANS: Automated network search
SE: Sensitivity
SP: Specificity
PPV: Positive predictive value
NPV: Negative predictive value
ROC: Receiver operating characteristics
AUC: Area under curve
CI: Confidence intervals
CT: Computerized tomography
MRI: Magnetic resonance imaging
ASIR: Age-standardized incidence rate
ASDR: Age-standardized death rate
DALYs: Disability-adjusted life years

## References

[1] Feigin VL, Stark BA, Johnson CO, Roth GA, Bisignano C, Abady GG (2021). Global, regional, and national burden of stroke and its risk factors, 1990–2019: A systematic analysis for the Global Burden of Disease Study 2019. Lancet Neurol..

[2] Pastore D, Pacifici F, Capuani B, Palmirotta R, Dong C, Coppola A (2017). Sex-genetic interaction in the risk for cerebrovascular disease. Curr. Med. Chem..

[3] World Health Organization. https://www.who.int/

[4] Fallahzadeh A, Esfahani Z, Sheikhy A, Keykhaei M, Moghaddam SS, Tehrani YS (2022). National and subnational burden of stroke in Iran from 1990 to 2019. Ann. Clin. Transl. Neurol..

[5] Bailey RR (2017). Promoting physical activity and nutrition in people with stroke. Am. J. Occup. Therapy.

[6] Kim HC, Choi DP, Ahn SV, Nam CM, Suh I (2009). Six-year survival and causes of death among stroke patients in Korea. Neuroepidemiology.

[7] United States Department of Health and Human Services. Centers for Disease Control and Prevention. https://www.cdc.gov/about/default.htm

[8] Assarzadegan F, Tabesh H, Shoghli A, Yazdi MG, Tabesh H, Daneshpajooh P (2015). Relation of stroke risk factors with specific stroke subtypes and territories. Iran. J. Public Health.

[9] Roach RE, Helmerhorst FM, Lijfering WM, Stijnen T, Algra A, Dekkers OM (2015). Combined oral contraceptives: The risk of myocardial infarction and ischemic stroke. Cochrane Database Syst. Rev..

[10] Xu Z, Li Y, Tang S, Huang X, Chen T (2015). Current use of oral contraceptives and the risk of first-ever ischemic stroke: A meta-analysis of observational studies. Thromb. Res..

[11] Lee PN, Thornton AJ, Forey BA, Hamling JS (2017). Environmental tobacco smoke exposure and risk of stroke in never smokers: An updated review with meta-analysis. J. Stroke Cerebrovasc. Dis..

[12] Zhu E, Chen Z, Ai P, Wang J, Zhu M, Xu Z (2023). Analyzing and predicting the risk of death in stroke patients using machine learning. Front. Neurol..

[13] Cheon S, Kim J, Lim J (2019). The use of deep learning to predict stroke patient mortality. Int. J. Environ. Res. Public Health.

[14] Rahman S, Hasan M, Sarkar AK (2023). Prediction of brain stroke using machine learning algorithms and deep neural network techniques. Eur. J. Electr. Eng. Comput. Sci..

[15] Zhang S, Wang J, Pei L, Liu K, Gao Y, Fang H (2021). Interpretability analysis of one-year mortality prediction for stroke patients based on deep neural network. IEEE J. Biomed. Health Inform..

[16] Çelik G, Baykan ÖK, Kara Y, Tireli H (2014). Predicting 10-day mortality in patients with strokes using neural networks and multivariate statistical methods. J. Stroke Cerebrovasc. Dis..

[17] Chung C-C, Chan L, Bamodu OA, Hong C-T, Chiu H-W (2020). Artificial neural network based prediction of postthrombolysis intracerebral hemorrhage and death. Sci. Rep..

[18] Amato F, López A, Peña-Méndez ME, Vaňhara P, Hampl A, Havel J (2013). Artificial neural networks in medical diagnosis. J. Appl. Biomed..

[19] Jiang F, Jiang Y, Zhi H, Dong Y, Li H, Ma S (2017). Artificial intelligence in healthcare: Past, present and future. Stroke Vasc. Neurol..

[20] Schwartz L, Anteby R, Klang E, Soffer S (2022). Stroke mortality prediction using machine learning: A systematic review. J. Neurol. Sci..

[21] Bishop. Neural Networks: A Pattern Recognition Perspective. https://www.microsoft.com/en-us/research/wp-content/uploads/1996/01/neural_networks_pattern_recognition.pdf (2023).

[22] Edmans J, Champion A, Hill L, Ridley M, Skelly F, Jackson T (2010). Occupational Therapy and Stroke.

[23] Das R, Turkoglu I, Sengur A (2009). Effective diagnosis of heart disease through neural networks ensembles. Expert Syst. Appl..

[24] Shakerkhatibi M, Dianat I, Asghari Jafarabadi M, Azak R, Kousha A (2015). Air pollution and hospital admissions for cardiorespiratory diseases in Iran: Artificial neural network versus conditional logistic regression. Int. J. Environ. Sci. Technol..

[25] Li L, Tang H, Wu Z, Gong J, Gruidl M, Zou J (2004). Data mining techniques for cancer detection using serum proteomic profiling. Artif. Intell. Med..

[26] Panzarasa S, Quaglini S, Sacchi L, Cavallini A, Micieli G, Stefanelli M (2010). Data Mining Techniques for Analyzing Stroke Care Processes. MEDINFO 2010.

[27] Shanthi D, Sahoo G, Saravanan N (2009). Designing an artificial neural network model for the prediction of thrombo-embolic stroke. Int. J. Biom. Bioinform. (IJBB).

[28] Amin, S. U., Agarwal, K. & Beg, R. (eds.) Genetic neural network based data mining in prediction of heart disease using risk factors. In *2013 IEEE Conference on Information and Communication Technologies* (IEEE, 2013).

[29] Kansadub, T., Thammaboosadee, S., Kiattisin, S. & Jalayondeja, C. (eds.) Stroke risk prediction model based on demographic data. In *2015 8th Biomedical Engineering International Conference* (*BMEiCON*) (IEEE, 2015).

[30] Lee E-J, Kim Y-H, Kim N, Kang D-W (2017). Deep into the brain: Artificial intelligence in stroke imaging. J. Stroke.

[31] Stathakis D (2009). How many hidden layers and nodes?. Int. J. Remote Sens..

[32] Knuiman MW, Vu HT (1996). Risk factors for stroke mortality in men and women: The Busselton Study. Eur. J. Cardiovasc. Prev. Rehabil..

[33] Easton JF, Stephens CR, Angelova M (2014). Risk factors and prediction of very short term versus short/intermediate term post-stroke mortality: A data mining approach. Comput. Biol. Med..

[34] Wang W, Jiang B, Sun H, Ru X, Sun D, Wang L (2017). Prevalence, incidence, and mortality of stroke in China: Results from a nationwide population-based survey of 480 687 adults. Circulation.

[35] Someeh N, Jafarabadi MA, Shamshirgaran SM, Farzipoor F (2020). The outcome in patients with brain stroke: A deep learning neural network modeling. J. Res. Med. Sci..

[36] Someeh N, Shamshirgaran SM, Farzipoor F, Asghari-Jafarabadi M (2020). The moderating role of underlying predictors of survival in patients with brain stroke: A statistical modeling. Sci. Rep..

